# Albendazole increases the inflammatory response and the amount of Em2-positive small particles of *Echinococcus multilocularis* (spems) in human hepatic alveolar echinococcosis lesions

**DOI:** 10.1371/journal.pntd.0005636

**Published:** 2017-05-25

**Authors:** Franz J. Ricken, Juliane Nell, Beate Grüner, Julian Schmidberger, Tanja Kaltenbach, Wolfgang Kratzer, Andreas Hillenbrand, Doris Henne-Bruns, Peter Deplazes, Peter Moller, Peter Kern, Thomas F. E. Barth

**Affiliations:** 1 Institute of Pathology, Ulm University, Ulm, Germany; 2 Division of Infectious Diseases, University Hospital and Medical Center, Ulm, Germany; 3 Department of Medicine I, University Hospital of Ulm, Ulm, Germany; 4 Department of General and Visceral Surgery, University Hospital of Ulm, Ulm, Germany; 5 Institute of Parasitology, University of Zürich, Zürich, Switzerland; Universidad Nacional Autonoma de Mexico, MEXICO

## Abstract

**Background:**

Alveolar echinococcosis (AE) is caused by the metacestode stage of *Echinococcus multilocularis*. The inflammatory response to this infection is influenced by the interaction of the parasite with the host. We aimed to analyze human liver lesions infected with *Echinococcus multilocularis* and the changes of the cellular infiltrates during albendazole (ABZ) treatment.

**Methodology/Principal findings:**

We analyzed liver tissue samples from 8 untreated patients, 5 patients treated with two daily doses of 400 mg ABZ for up to two months and 7 patients treated for more than two months with the same ABZ therapy. A broad panel of monoclonal antibodies was used to characterize the lesion by immunohistochemistry. A change in the cellular infiltrate was observed between the different chemotherapy times. During the initial phases of treatment an increase in CD15^+^ granulocytes and CD68^+^ histocytes as well as in small particles of *Echinococcus multilocularis* (spems) was observed in the tissue surrounding the metacestode. Furthermore, we observed an increase in CD4^+^ T cells, CD20^+^ B cells and CD38^+^ plasma cells during a longer duration of treatment.

**Conclusions/Significance:**

ABZ treatment of AE leads to morphological changes characterized by an initial, predominantly acute, inflammatory response which is gradually replaced by a response of the adaptive immune system.

## Author summary

Alveolar echinococcosis (AE) is a life-threatening disease in humans caused by the larval stages of *E*. *multilocularis*. It has been shown that the infection in humans is associated with a modulated immune response. Depending on multiple factors, such as the stage of disease, total or partial surgical resection and albendazole (ABZ) therapy are treatments of choice. ABZ is known as a parasitostatic drug that has to be administered for years to suppress metacestode development. Here we compared human liver lesions before and after short and long term treatment with ABZ by immunohistochemistry using a broad panel of antibodies. We found a change in the cellular infiltrate, characterized by a shift to an infiltrate rich in T cells, B cells and plasma cells during long-term treatment with ABZ, including a pronounced detection of small particles of *E*. *multilocularis* (spems). We argue that ABZ treatment is likely to change the cellular infiltrate, leading to an enhancement of the host immune response during treatment.

## Introduction

Human alveolar echinococcosis (AE), caused by the tapeworm *Echinococcus multilocularis*, is considered one of the most pathogenic zoonosis in humans with endemic areas in the Northern hemisphere and also in Western China [1,2]. The adult worms live in the intestine of canids, such as the red fox (*Vulpes vulpes*). Eggs are released into the environment through the feces of canids. In humans who accidentally ingest the eggs, the multi-vesicular metacestode shows a tumor-like growth pattern predominantly in the liver. However, the disease may spread to other organs [3]. If possible, first-line treatment is radical surgery, accompanied by treatment with benzimidazole derivatives. Lifelong treatment is necessary if the patient has non-resectable lesions.

The metacestode stage of *E*. *multilocularis* consists of a cellular germinal layer surrounded by an acellular laminated layer. The laminated layer synthesized by the germinal layer is the histological hallmark of the lesion [4]. Since the laminated layer is rich in polysaccharide protein complexes, these fragments have a high affinity to PAS staining and are well recognized on histological examination. The central core of the lesion is necrotic and may contain particles of protoscoleces and fragments of the laminated layer [5]; this zone is surrounded by a cellular infiltrate [6].

The monoclonal antibody Em2G11 is specific for the Em2 antigen of the *E*. *multilocularis* metacestode and exclusively stains the laminated layer as well as the cyst content in tissue sections. Additionally, the antibody marks acellular Em2-antigen-positive small particles of *E*. *m**ultiloculari**s* (spems) inside and outside the main lesion [7]. These particles are probably shed due to the growth of the metacestode and/or the inflammatory response [8] and may play a modulatory role in the immunological process during the infection [9].

Infection with *E*. *multilocularis* in humans is characterized by modulation of the immune response, which allows the parasite to escape the immune response of the host [10],[11]. This phenomenon is reflected by changes in the cytokine profile and the T-helper cell response. During the course of inflammation, the acute inflammatory Th1 response is gradually converted into a Th2 response in mice, reflecting the chronic phase of AE [12,13].

The severity of the disease may depend on the genetic background of the host and on the acquired disturbances of the Th1-related immunity [12,14,15]. The laminated layer of the metacestode, particularly its carbohydrate components, plays a major role in the evasion of cellular and humoral immunomechanisms and, furthermore, in tolerance induction and immunomodulation [16]. The Em2 antigen is a T cell-independent antigen and the response against Em2 antigen has been shown to lack antibody maturation [9]. Moreover, in contrast to Em492 antigen, the Em2 antigen does not lead to anti-CD3 apoptosis. Em492 stimulates peritoneal macrophages to produce high levels of nitric oxide leading to an inhibition of murine splenocyte proliferation [11], therefore acting as an immunosuppressant [17].

Th2-type and anti-inflammatory cytokines, IL-10 and TGF-*β*, as well as nitric oxide, are involved in the maintenance of tolerance and partial inhibition of cytotoxic mechanisms [12,18]. The complex immune response during infection is characterized by an abnormal production of various interleukins, such as interleukin-5 [19,20], IL-27 [21], high levels of IL-10 and TGF-beta [18,22]. Simultaneously, insufficient production of IL-12 [23], IL-31, IL-33 [21], IFN-gamma and cytotoxic T cells leads to an enhanced tolerance towards the parasite. It has been shown that the Fibrinogen-like-protein 2 (FGL2), a CD4^+^CD25^+^ regulatory T cell effector molecule secreted by T regulatory cells, plays a crucial role in the immune response by suppressing Th1 and Th17 responses [24]. In line with this observation, mice infected with *E*. *multilocularis* eggs showed up-regulation of FGL2 in the liver [25].

Long-term treatment with ABZ has improved the 10-year survival rate in comparison with untreated historical controls from 6–25% to 80–83% [26][27].

ABZ binds to beta-tubulin and inhibits absorptive functions in *E*. *multilocularis* [28]. ABZ also binds to beta-tubulin in the human host, which is very similar with more than 90% identical amino acids between the parasite and humans [29]. Treatment results in an inhibition of metacestode proliferation, and leads to destruction of protoscoleces; it inhibits formation of the germinal layer and therefore of the metacestode [27]. ABZ treatment is regarded as parasitostatic [30], [31]; in some cases benzimidazoles show a parasitocidal action [32] *in vitro*.

Here, we hypothesized that treatment with ABZ may have an influence on the cellular infiltrate of *E*. *multilocularis* in infected human liver tissue. Therefore, we conducted a morphological and immunohistological analysis of 20 human liver tissue lesions (12 treated/8 untreated with ABZ) using a broad panel of antibodies to characterize the lesions.

## Materials and methods

### Ethics statement

The study has been approved; see: Zentrale Ethikkommission bei der Bundesärztekammer. Mitteilungen: Die (Weiter-) Verwendung von menschlichen Körpermaterialien für Zwecke medizinischer Forschung. Dtsch Arztebl. 2003: 1632.

### Patients and tissue samples

Human liver tissue samples from 20 patients were retrieved from the paraffin archives of the Institute of Pathology, University of Ulm. The samples were anonymized according to German law for correct usage of archival tissue for clinical research [33]. Of the 20 liver specimens with histologically confirmed *E*. *multilocularis* infection, 8 samples are from untreated patients (patients #1–8), 5 samples from patients treated with 2 x 400 mg ABZ (Eskazole) per day for up to two months (patients #9–13) and 7 patients treated for more than two months with 2 x 400 mg ABZ (Eskazole) per day (patients # 14–20). 17 cases were resection samples and 5 cases were cutting needle biopsies with more than 90% of representative tissue of the lesion. Cutting needle biopsies were performed as a diagnostic step regarding a liver lesion of unknown significance. The clinical characteristics of the patients are shown in Table 1. The cohort was divided into three groups: group 1 = no therapy, group 2 = treatment of up to two months with a range between 4 and 37 days’ treatment, group 3: treatment of more than two months. These groups were formed on the basis of samples of patients available for the analysis.

**Table 1:** Characteristics of the patients.

No.	Age at surgery	Sex	Organ/Site	Type of lesion	Material	Medication/dose	Albendazole treatment time before surgery
1	82	F	liver	4–5 lesions, largest lesion 79x61x70mm	biopsy	/	/
2	56	M	liver	not specified, incidental finding during surgery inguinal hernia	partial hepatectomy	/	/
3	65	F	peritoneum	5 lesions, largest lesion 34x30mm	biopsy	/	/
4	54	M	liver	1 lesion, size 37x28x32mm	biopsy	/	/
5	66	M	liver	1 lesion, size 96x88mm	biopsy	/	/
6	61	F	liver	n.a.	partial hepatectomy	/	/
7	60	F	liver	n.a.	partial hepatectomy	/	/
8	47	F	liver	n.a.	partial hepatectomy	/	/
9	68	F	liver	1 lesion, size 28x32mm	partial hepatectomy	Albendazole/800 mg	4 days
10	63	F	liver	1 lesion, size 148x136mm	partial hepatectomy	Albendazole/800 mg	19 days
11	49	F	liver	multiple lesions, largest lesion 80x90mm	partial hepatectomy	Albendazole/800 mg	27 days
12	41	M	liver	1 lesion, size 100x60mm	partial hepatectomy	Albendazole/800 mg	36 days
13	29	F	liver	1 lesion, size 37x34x72mm	partial hepatectomy	Albendazole/800 mg	37 days
14	74	F	liver	1 lesion, size 83x70x77mm	partial hepatectomy	Albendazole/800 mg	intermittent 3 months (total approximately 60 days)
15	53	M	liver	2 lesions, largest lesion 54x40mm	partial hepatectomy	Albendazole/800 mg	105 days
16	47	F	liver	1 lesion, size 53x44mm	partial hepatectomy	Albendazole/800 mg	125 days
17	20	F	liver	3 lesions, largest lesion 45x32mm	partial hepatectomy	Albendazole, 107 days 800 mg, 57 days 400 mg	164 days
18	72	M	liver	3 lesions, largest lesion 37x30x25mm	partial hepatectomy	Mebendazole/ 1500 mg 85 days, Albendazole, 800mg 132 days	intermittent 9 months (total approximately 217 days)
19	60	F	liver	2 lesions, largest lesion 66x59x40mm	partial hepatectomy	Albendazole/800 mg	2 years
20	48	F	liver	1 lesion, size 97x60x70mm	partial hepatectomy	Mebendazole, Albendazole/800 mg	intermittent 18 years

### Staining procedures

Hematoxylin and eosin (HE) and Periodic Schiff staining (PAS) staining, as well as immunohistochemistry, were performed according to standard protocols [7].

The resection specimens and biopsies were fixed in 4% buffered formaldehyde for at least 36 hours. Serial sections of about 3 μm from paraffin blocks with representative tissue were performed with a microtome. Paraffin was dissolved with xylol and ethanol.

For antigen retrieval, different pretreatment methods were used according to the companies’ recommendation. As primary antibodies, the following antibodies were used: monoclonal antibody CD3 (F7.2.38, 1:100 dilution, DAKO, Glostrup, DK), CD4 (4B12, 1:200 dilution, DAKO), CD8 (C8/144B, 1:200, DAKO), CD15 (MMA, 1:300, BD; Erembodegem, BEL), CD20 (L26, 1:500, DAKO), CD38 (SPC32, 1:100, Menarini, Florence, IT)), CD68 (PG-M1, 1:100, DAKO), eosinophil major basic protein (EMBP) antibody (BMK-13, 1:25, Zytomed Systems; Berlin, GER), Em2G11 (1:100; kind gift of Peter Deplazes, Institute of Parasitology, University of Zürich, Switzerland) and FGL2 (1:4000, Abnova; Taipeh, TW).

The primary antibody was diluted in Antibody Dilution solution (DAKO) and each slide was incubated with 50 μl in a humid chamber at room temperature for 30 min. The DAKO REAL Detection System, Alkaline Phosphatase/Red (DAKO, Carpintera, CA, USA) was used as the detection system according to the manufacturer’s protocols. As negative controls, staining was performed without the primary antibody.

The evaluation of the immunohistological stainings was carried out in a blinded fashion by three observers (TFEB; FJR; JN) at a multihead microscope. Five different high-power observation fields of one section centering on the inflammation zone between the normal liver parenchyma and the necrosis were analyzed and the stained cells counted (400x magnifications). The average for each section was calculated. Regarding the possible sample error, we stained representative sections of two different blocks of tissue of three cases with the whole antibody panel. In sections of two cutting needle biopsies, only four positions were evaluated. To measure the quantity of spems, we determined the percentage of the whole necrotic area with a typical pattern of spem staining using a 25x magnification. To register the lymphatic aggregates, we counted all lymphatic aggregates larger than 1 mm within an area of 1.2 cm in diameter.

The average and the standard deviation for each evaluation, was calculated. Furthermore, we performed a two-sided t-test type 3 with unequal variance with Excel (Microsoft Office 2007) and IBM SPSS (Statistic Version 21, IBM Corp.). The result was regarded as significant for p-values p< 0.05.

## Results

Using HE staining, we first defined the microscopical parameters of the lesion. All lesions, with and without treatment with ABZ, had a central necrosis of varying diameters in common; next followed an inner circle close to the necrotic zone, characterized by epithelioid cells and granulocytes, and an outer circle with lymphocytes followed by hepatic tissue. Between the outer and the inner zone, a fibrotic layer of varying diameter was found. Of note, fragments of protoscoleces were found only once in 20 samples (case # 10).

On analysis of the different parameters of the inflammatory infiltrate, histological differences and similarities were noted.

All lesions had the following immunohistological characteristic in common: In a CD68 staining, the macrophages and epithelioid cells were highlighted in the inner zone. CD15^+^ granulocytes were intermingled with the CD68^+^ cells in the inner circle but not in the outer circle. Some positive EMBP eosinophilic granulocytes were resident in the inner zone.

The outer circle was characterized by a mixture of CD8^+^ and CD4^+^ T cells; CD8^+^ cells were generally more frequent than CD4^+^ cells. CD20^+^ B cells were mixed with the T cells. CD38^+^ plasma cells were interspersed predominantly in the outer zone.

**Fig 1:**
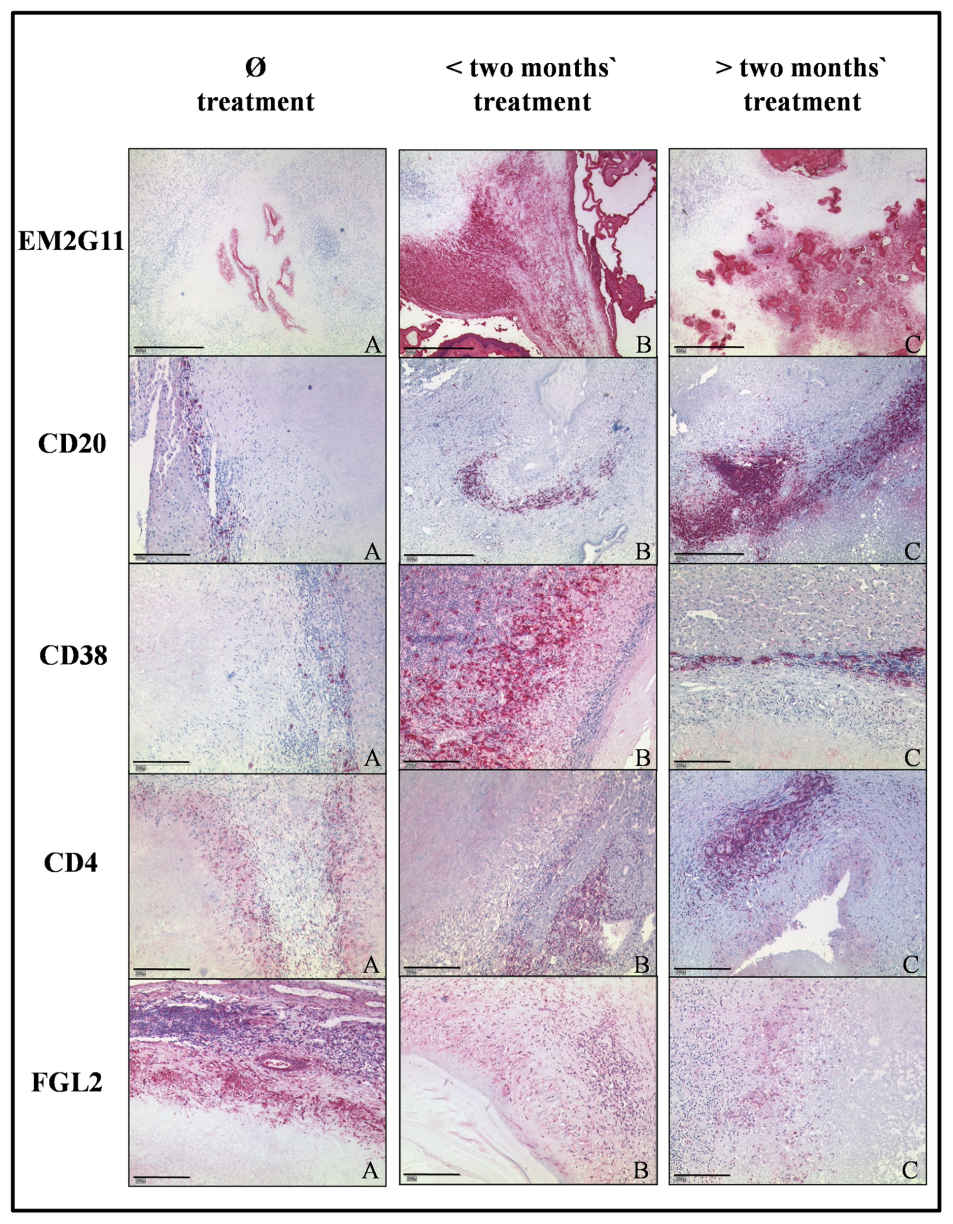
Immunohistochemical analysis of sections of liver tissue from patients with *E*. *multilocularis* infection without ABZ treatment (n = 8), < two months’ (n = 5) and > two months’ treatment with ABZ (n = 7). EM2G11 staining shows an increase in spems from sections without treatment as compared to stainings of samples with treatment up to two months and a decrease in > two months’ treatment. (A: Case #3, B: Case #11, C: Case #19, bar = 500μm) CD20 staining illustrates an increase in B cells during treatment. (A: Case #7, B: Case #12, C: Case #19, bar = A: 200μm, B, C: 500μm) CD38 staining shows an increase in plasma cell content with a maximum in < two months of ABZ treatment. (A: Case #6, B: Case #9, C: Case #17, bar = 200μm) CD4 staining reveals an increase in the number of CD4^+^ T cells in the tissue after treatment for > two months. (A: Case #3, B: Case #13, C: Case #18, bar = 200μm) FGL2 staining shows a decrease in the number of FGL2^+^ cells with a minimum at < two months of treatment. (A: Case #7, B: Case #12, C: Case #18, bar = 200μm).

**Fig 2:**
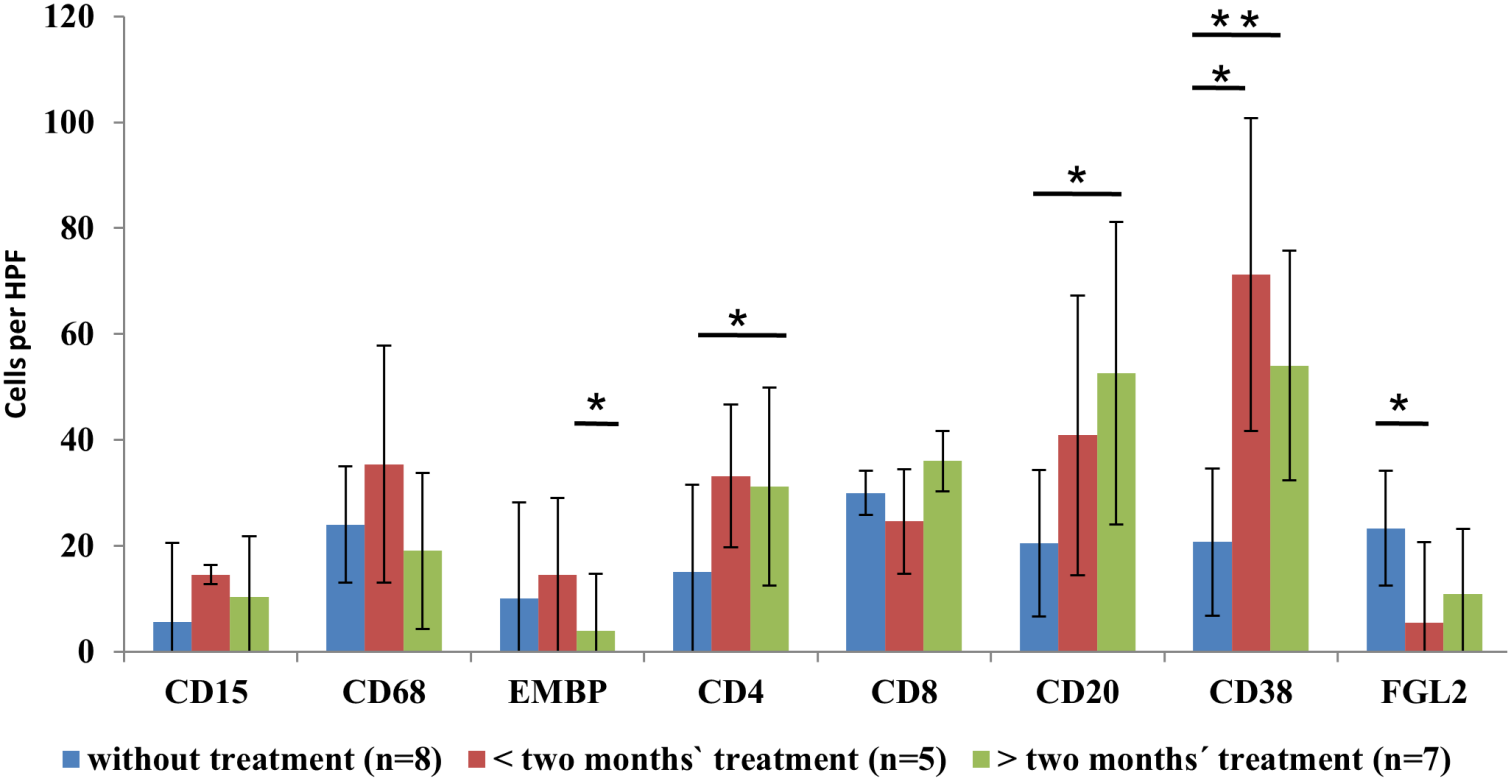
Detection values of different antigens tested without (n = 8), < two months (n = 5) and > two months of treatment (n = 7) with ABZ in human alveolar echinococcosis liver lesions (* = p < 0.05, ** = p < 0.01, HPF = High power field).

Regarding the time course of ABZ treatment, we noted differences in the composition of the cellular infiltrate, which varied in relation to the duration of treatment. In the lesions of patients treated up to two months, CD68^+^ and CD15^+^ cells were more prominent in the inner zone compared to non-treated lesions and lesions treated for over two months. With respect to T cells, CD4^+^ cells increased significantly and the number of CD8^+^ cells remained largely stable during the course of treatment. CD20^+^ B cells and CD38^+^ plasma cells increased significantly (Figs 1 and 2, S1 Table) with plasma cells outnumbering B cells. Along with the increased number of T and B cells, we noted an increase in lymph follicles larger than 1 mm consisting of CD4^+^/CD8^+^ T cells and CD20^+^ B cells. Small particles of *E*. *multilocularis* (spems) increased during the course of treatment. Spem staining was most prominent during the initial treatment phase up to two months (Fig 1). Spems were detected in the necrotic area, in sinusoids, vessels and lymph follicles around the lesion. In contrast, FGL2^+^ cells showed a tendency to decrease under therapy, being lowest in patients having a short duration of treatment (Fig 1).

**Fig 3:**
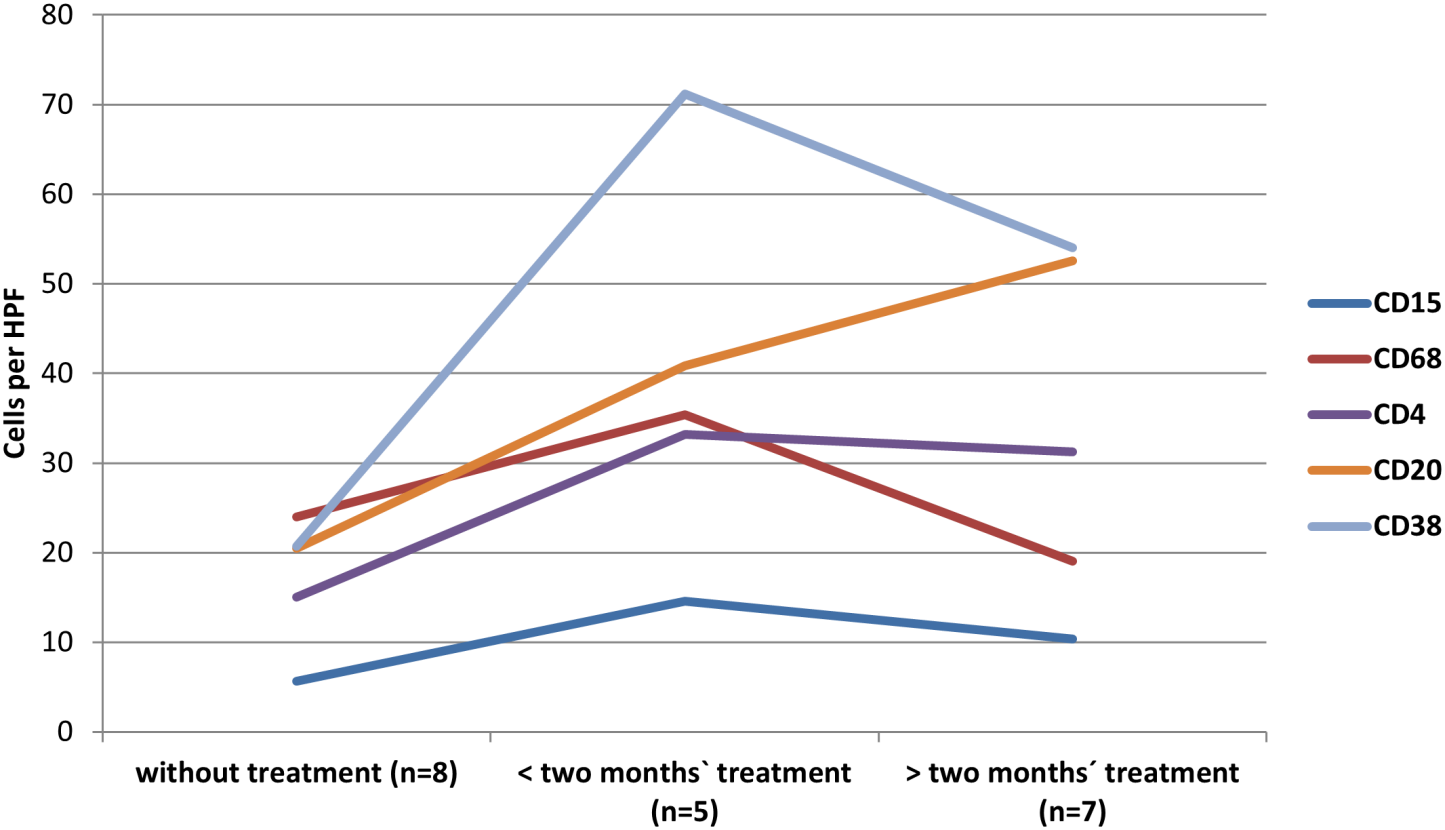
Summary of expression of various antigens in human liver lesions with *E*. *multilocularis*. There is an increase in CD15^+^ and CD68^+^ cells during the first two months of treatment, which then diminishes. CD38^+^ and CD4^+^ cells increase during the first two months and then reach a plateau. CD20^+^ cells show a constant increase during treatment.

These results show that the inflammatory infiltrate changes during early and late treatment with an increase in macrophages and granulocytes during the first six weeks of treatment followed by a shift to a specific cellular response with an increase in CD4^+^ T cells during early response; by contrast, the number of CD8^+^ T cells remains stable during treatment. Furthermore, CD20^+^ B cells, plasma cells and lymph follicles generally increase during late treatment (Fig 3). Immunohistological analysis of sections of two different paraffin blocks from one resection specimen from the same patient showed almost identical immunohistological results. This was repeated for two samples from three patients and therefore confirmed the method. The cohorts were also analyzed with a cut-off level of one month. The results showed the same trends as described for the two-months threshold (S1 Fig).

## Discussion

To date, only very few histological/immunohistological studies characterizing the inflammatory infiltrates around the human lesions of *E*. *multilocularis* before and after treatment have been performed [34,35]. We detected a morphological spectrum of lesions caused by an infection with *E*. *multilocularis* which is characterized by irregular sized metacestodes [36] and the lamellar layer, which is the hallmark of these lesions [7]. We detected protoscolex remnants in only one sample, which is in agreement with the literature (less than 10%) [3,37]. Based on our morphological and immunohistological data, the lesion is characterized by an inner layer with a cellular composition typical of a non-specific response consisting of macrophages and granulocytes and an outer layer consisting of T and B cells.

This confirms data from mice infected with *E*. *multilocularis*, which showed an intense granulomatous infiltration in the periparasitic area of the lesions [37].

Peripheral blood mononuclear cells and polymorph nuclear granulocytes are activated after stimulation *in vitro* with *E*. *multilocularis* vesicles and synthesize interleukin-8 (IL-8) [38] and monocyte chemo attractant protein-1 (MCP-1) [39]. IL-8 leads to neutrophil migration and activation [40] and MCP-1 attracts and activates macrophages [41] and is an attractant for CD4^+^ and CD8^+^ T cells. These findings are reflected by our data showing that T cells are present in the lesions *in situ* and are increased during ABZ therapy.

In mice infected with *E*. *multilocularis*, the number of CD4^+^ and CD8^+^ cells is reduced, probably due to the diminished ability of antigen-presenting cells to present conventional antigens [42]. Furthermore, there is an elevation in CD4^+^ T cells in abortive or died-out lesions and active metacestodes are indicated by higher levels of CD8^+^ T cells [34]. ABZ acts as an intracellular tubulin inhibitor [28] and prevents metacestode formation. In mice, treatment with ABZ leads to loss of integrity in the germinal layer and a reduction in tumor mass [43]. Liance et al. [37] showed that rodents inoculated with *E*. *multilocularis* material from treated human patients have a decreased larval development in contrast to inoculation with samples from untreated patients. At a high concentration, ABZ leads to a collapse of the alveolar architecture of the parasite, partially dissolving the laminated layer followed by an invasion of the lesion with host inflammatory cells, such as histocytes, lymphocytes, neutrophils and eosinophils [44].

Reduction of the width of the laminated layer upon therapy [45] was confirmed in our study and degradation of the laminated layer may contribute to the observed increase of spems in and around the lesion, such as sinusoids, vessels and lymph follicles, which may influence the immune reaction [7]. In support of our immunohistological finding ABZ treatment has been shown to affect differentiated cells of *E*. *multilocularis* including the tegument, which is responsible for the production of the laminated layer [46]; therefore, it might be hypothesized that by this mechanism ABZ treatment leads to an increased immunohistological detection of spems.

Taken together, we found an overall increase in the number of immune cells during the course of treatment with ABZ. This effect was enhanced in the first weeks of treatment with ABZ. Our findings support the view that the non-specific immune reaction is activated at the beginning of treatment with an increase in macrophages and granulocytes, which then reduce during later treatment; our data suggest that this response is shifted towards the specific immune response, dominated by B and plasma cells which, however, do not eliminate the infection. Therefore, ABZ treatment supports the activation of the host immune system by reducing the immunosuppressive functions of the parasite. Our data suggest that, by reducing the metabolism of the metacestode during ABZ treatment and dissolution of the laminated layer, more parasite antigens are exposed and detected by the immune system and that this may lead to a more specific immune response. Supportive of this finding is that protoscoleces not protected by the laminated layer are killed by macrophages [47]. Furthermore, there are several parasite excretory/secretory products with suppressive effects on the immune system of the host [48]; by damaging the tegument, the function of these products may be reduced and may, in turn, lead to an increase in the immune response of the host.

FGL2, secreted by macrophages and T regulatory cells, leads by various mechanisms to an suppressed immune status of the host and to a progression of the metastatic growth [49]. It has been shown in mice that FGL2 suppresses the Th1 and Th17 immune response and supports the Th2 response [24]. Our finding that the FGL2 effector molecule is reduced during ABZ treatment corresponds to these observations. This indicates that treatment with ABZ may lead to a change in the immune response towards a Th1-shifted immune response by down-regulation of FGL2.

To summarize, our histological study confirms and extends findings of *in vitro* and *in vivo* studies in mice and humans infected with *E*. *multilocularis* and may help to explain the mechanism of action of ABZ during the course of treatment of patients with an initial acute inflammatory response that is gradually replaced by the adaptive immune system. The finding that spems are increased during early treatment may point to a role of spems as mediators of this inflammatory response.

## Supporting information

S1 Table Comprised raw data of analyzed values. (The mean value and in brackets the range of values).
(TIF)

S1 Fig Detected values of different antigens tested without treatment (n = 8), < one month (n = 3) and > one month of treatment (n = 9) with ABZ in human alveolar echinococcosis liver lesions (* = p < 0.05, ** = p < 0.01, HPF = High power field). (TIF)
