# Supplementary figures and images for: Albendazole increases the inflammatory response and the amount of Em2-positive small particles of Echinococcus multilocularis (spems) in human hepatic alveolar echinococcosis lesions
Source: PLoS Negl Trop Dis. 2017 May 25;11(5):e0005636. doi: 10.1371/journal.pntd.0005636 (PMC5462468; doi:10.1371/journal.pntd.0005636)

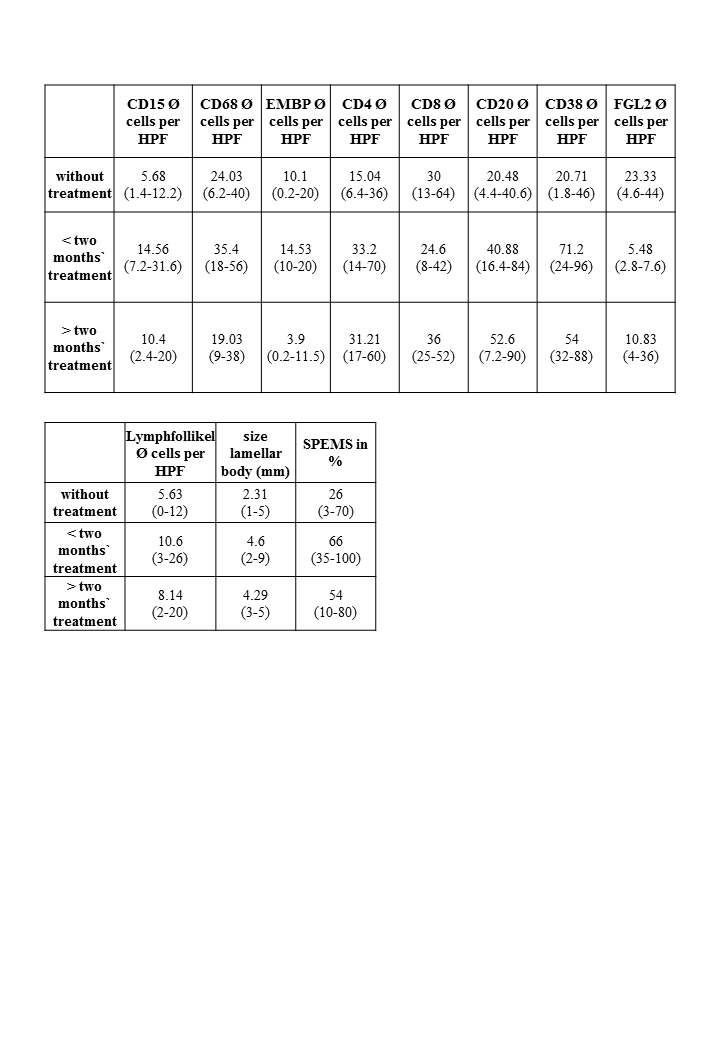

Supplement: S1 Table — (The mean value and in brackets the range of values). (TIF) [file pntd.0005636.s001.tif]

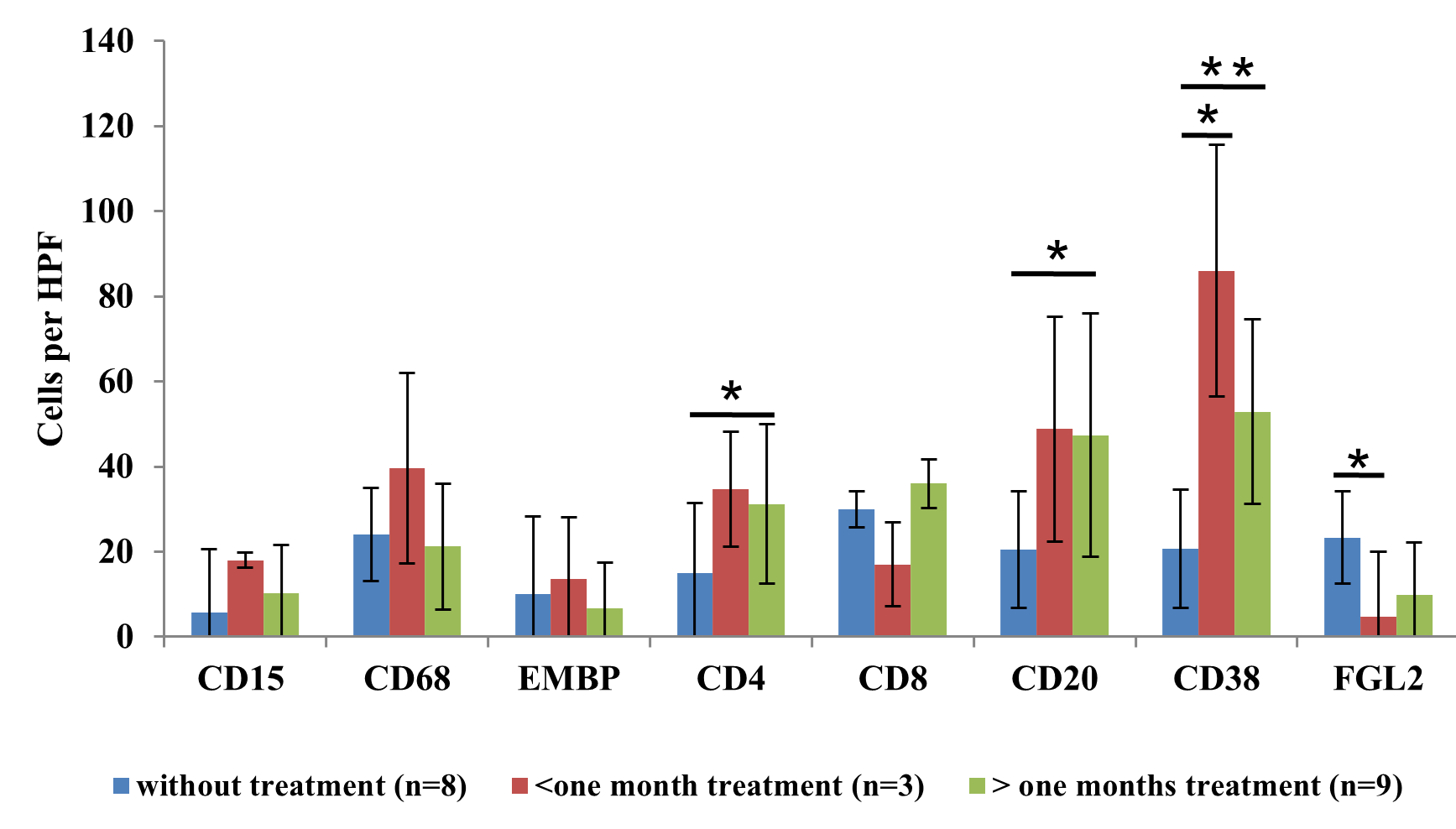

Supplement: S1 Fig — (TIF) [file pntd.0005636.s002.tif]
